# Clinical characteristics and outcomes after shunt surgery in idiopathic normal pressure hydrocephalus with or without prior cerebrospinal fluid tap testing: a single-center follow-up study of 481 patients

**DOI:** 10.1186/s12987-026-00802-9

**Published:** 2026-03-31

**Authors:** Kardelen Akar, Lena Kollen, Hanna C. Persson, Mats Tullberg

**Affiliations:** 1 Department of Clinical Neuroscience, Institute of Neuroscience and Physiology, Sahlgrenska Academy, University of Gothenburg, Gothenburg, Sweden; 2 Graduate School of Health Sciences, Koç University, Istanbul, Türkiye; 3 Department of Occupational Therapy and Physiotherapy, Sahlgrenska University Hospital, Gothenburg, Sweden; 4 Department of Neurology, Sahlgrenska University Hospital, Gothenburg, Sweden

**Keywords:** Idiopathic normal pressure hydrocephalus, Hydrocephalus, Normal pressure, Cerebrospinal fluid, Tap test, Outcome assessment, Cerebrospinal fluid shunts, Gait, Postural balance

## Abstract

**Background:**

The cerebrospinal fluid tap test (CSF TT) is a commonly used predictive test for selecting patients with idiopathic normal pressure hydrocephalus (iNPH) for shunt surgery, but low sensitivity rate carries the risk of excluding individuals from effective treatment. We explored clinical characteristics and postoperative outcomes of iNPH patients shunted based solely on clinical and MRI findings compared with those shunted following a positive CSF TT.

**Methods:**

A total of 481 consecutive shunt operated iNPH patients were assessed in a team-based setting. Patients were categorized into two groups: those shunted based on typical clinical symptoms and MRI features without a supplementary test (NoTT, *n* = 390) and those shunted based on a positive CSF TT (TT, *n* = 91). Baseline clinical data, including comorbidities, and 5-month postoperative outcomes were assessed using the Gothenburg iNPH Scale and the modified Rankin scale (mRS).

**Results:**

Baseline characteristics and clinical measures were similar across groups, except that TT patients had more prevalent other concurrent neurological conditions (37% vs. 11%) and a longer delay between diagnosis and surgery (median 198 vs. 126 days) (both *p* < 0.001). Overall improvement (≥ 5-point postoperative increase in the total iNPH Scale) was observed in 70.9% of NoTT patients compared with 58.6% of TT patients (percentage point difference = 12.3; 95% CI = 0.3, 24.3; *p* = 0.044) while postoperative deterioration (defined as ≥ 5-point decrease on the iNPH Scale) was significantly more common in TT group (22.9%) than in the NoTT (10.7%) (percentage point difference = 12.1; 95% CI = 3.5, 20.7; *p* = 0.006). After adjusting for concurrent neurological disorder and surgical delay, improvement in continence (percentage point difference = 13.5, 95% CI = 0.7, 26.3) was better in NoTT patients (*p* = 0.039).

**Conclusions:**

This real-world study shows that a majority of iNPH patients can be routinely assessed by a clinical team and shunted with favorable outcomes without a supplementary predictive tap test. The worse outcomes seen in TT patients are likely to be due to higher prevalence of neurological comorbidity and delayed time to surgery. We suggest that the tap test may be reserved for difficult patients with suspected iNPH including those who present with concurrent other neurological disorders.

**Supplementary Information:**

The online version contains supplementary material available at 10.1186/s12987-026-00802-9.

## Introduction

Idiopathic Normal Pressure Hydrocephalus (iNPH) is a reversible neurological disorder, characterized by enlarged ventricles and impaired gait and balance, cognitive decline, and urinary incontinence, and is common in people aged 65 or above [1–3]. According to international guidelines, the diagnosis of iNPH relies on a combination of typical clinical symptoms, characteristic features on brain computed tomography (CT) or magnetic resonance imaging (MRI), and a normal cerebrospinal fluid (CSF) pressure [1, 4]. CSF diversion via shunt surgery is considered a cost-effective treatment, resulting in clinical improvement in up to 80% of patients [5–7].

Supplementary tests evaluating CSF hydrodynamics, such as the cerebrospinal fluid tap test (CSF TT), extended lumbar drain (ELD) or lumbar infusion test, are commonly used to predict shunt surgery outcomes, and thus inform the decision to recommend surgery or not [8, 9]. While these tests demonstrate high positive predictive values [10–14], their relatively low negative predictive values introduce a risk of excluding patients who might benefit from shunt surgery. It is therefore advocated that these tests be used as complementary tools rather than as stand-alone criteria for selecting shunt candidates [8, 12, 13].

The Japanese guidelines [15], which show compatibility with neurologists’ clinical judgement [16], recommend shunt surgery for patients presenting with gait disturbances, enlarged ventricles, and MRI features of tight high convexity and medial subarachnoid spaces, and disproportionally enlarged subarachnoid space hydrocephalus (DESH), in combination with normal CSF pressure and normal CSF analysis [4]. However, reliance on DESH has been questioned due to its limited diagnostic accuracy and low prevalence among iNPH patients [17]. To date, there is no established standard for prognostic evaluation in iNPH, and it remains unclear how to identify patients who are eligible for additional testing early in the clinical work-up [10], an ambiguity that has been highlighted in research. Recently, in a randomized placebo-controlled study on shunt efficacy in iNPH, defined by gait velocity improvement, only patients showing with a positive CSF drainage test were included [18]. Conversely, in the European multicenter trial on iNPH, patients were selected for surgery solely based on clinical symptoms and MRI-findings, with favorable 12-months outcomes observed in 84% of patients, as measured by improvements across iNPH Scale domains [5].

For many years, our center—the sole adult hydrocephalus referral center serving the 1.7 million inhabitants of the Western Sweden region—has employed a multiprofessional, team-based diagnostic algorithm encompassing the entire process of diagnosis, treatment, and follow-up. Patients with typical clinical assessed symptomatology, along with supportive MRI and CSF findings, are considered eligible for shunt surgery without the need for additional predictive testing. Supplementary tests such as the CSF tap test and lumbar infusion test are reserved for cases where the diagnosis is uncertain, most often due to unclear or atypical symptoms [2, 19–21]. This rigorous assessment process aims to efficiently manage high-certainty cases while ensuring that shunt responders are not missed. Using the Gothenburg iNPH Scale introduced by Hellström [22] to assess postoperative outcomes, this approach has consistently reported high response rates overall in iNPH patients [2, 5, 23, 24].

The aim of this study is to investigate potential differences in baseline clinical characteristics and postoperative outcomes in a consecutive real-life sample of iNPH patients who were shunted based on typical clinical symptoms and MRI features alone compared to patients who were shunted after a prior positive CSF TT.

## Methods

### iNPH diagnosis and patient selection

This single-center, longitudinal cohort study included 481 consecutive patients diagnosed with iNPH according to international guidelines [1] and subjected to shunt surgery between the 1st of January 2017 and 31st December 2023 at the Hydrocephalus Research Unit, Sahlgrenska University Hospital, Sweden. All patients referred to the Hydrocephalus Research Unit for diagnostic work-up of suspected iNPH during the time were eligible for inclusion. Patients who had their shunt surgery later than August 31st of 2024 or who were unable to undergo the standardized physiotherapist assessment of gait and balance both pre- and postoperatively were excluded.

### Diagnostic procedures

All patients underwent a standardized team-based assessment, including clinical examinations and tests performed by a neurologist, a physiotherapist, a neuropsychologist, in addition to an MRI of the brain at baseline and postoperatively, as described elsewhere [24]. A lumbar puncture (LP) with measurement of opening pressure, analysis of CSF cell count, protein content and biomarkers of neurodegeneration as well as routine blood laboratory tests were performed at baseline.

Following these assessments, a weekly multiprofessional team round table discussion comprising neurologists, nurses, physiotherapists, neuropsychiatrists and neurosurgeons was conducted to review findings in a holistic approach. Based on this review, patients were either referred directly to shunt surgery (NoTT), or when the surgical outcomes were claimed uncertain mainly due to atypical or unclear symptoms and/or MRI findings, a supplementary CSF TT was used to strengthen the indication for shunt operation (TT). In such cases, only patients showing improvement at the CSF TT were operated upon.

All patients received a Strata valve (Medtronic, Goleta, USA) set at baseline pressure of 1.5 cm of water. All patients were followed up post operatively using the same clinical and MRI protocols. Shunt function was assessed by evaluation of clinical symptoms and CT or MRI as earlier reported [24]. If doubts regarding shunt patency remained following clinical testing and CT or MRI, i.e. lack of a clear postoperative clinical improvement and/or clear postoperative change on CT or MRI, a radionuclide shuntography [25] and/or a lumbar infusion test was performed. All shunts were deemed working at the time of follow up.

### Baseline MRI measurement

MRI of the brain was performed at baseline using standardized protocol including Fluid Attenuated Inversion Recovery (FLAIR), T1-weighted, Diffusion Weighted Imaging (DWI), and sagittal flow-sensitive sequences. MRI images were assessed by trained neuroradiologists according to a standard protocol as part of the routine radiological workup starting in 2020, utilizing axial, coronal and sagittal 3D images. Radiological measures assessed were Evans’ index, Callosal angle, mean temporal horn width (mm). Presence of tight high convexity sulci (THC), Sylvian fissure dilatation, and infarctions were recorded as binary variables (yes/no). To assess Disproportionally Enlarged Subarachnoid Space Hydrocephalus (DESH) [26], concurrent presence of both THC and Sylvian fissure dilatation (yes/no) was registered. White matter hyperintensity was graded using Fazekas scale (0–3), where a higher number represents more extensive white matter changes [27].

### Clinical measures and outcomes

At the initial clinical visit demographic data, including age, sex, body mass index (BMI), comorbidities, and disease duration were collected.

The pre- and postoperative clinical ratings utilized the Gothenburg iNPH Scale introduced by Hellström [22] where the total score is the mean of four domain scores: gait, balance, cognitive function, and continence with gait rated twice. A score of 0 represents maximum symptom burden and 100 equals the performance of a healthy 70-year-old.

Postoperative outcome was calculated as the change between the post-and the preoperative scores. Patients were categorized as “Improved” if their postoperative score had increased by at least five points, “Unchanged” if the change was between − 4 and 4 points, and “Deteriorated” if the score was decreased by at least five points [22].

The modified Rankin Scale (mRS) [28] (0–6 points, higher scores indicating worse function) was used to assess functional disability and dependence, and the Mini Mental State Examination (MMSE) [29] (0–30 points, higher scores indicating better function) to assess global cognitive function.

### TT group

The TT group underwent a standard CSF TT procedure, modified from Wikkelsö et al. [11], including comprehensive assessment of gait, balance and cognitive function. Patients were scheduled for a two-day clinical evaluation. On day 1 at 2:00 PM, the baseline (pre-tap) assessment comprised standardized comprehensive physiotherapy tests, including the 10-meter walk test at both self-selected and maximum speed (recording time in seconds and step count), the Timed-Up and Go Test (seconds and steps), the 3-meter backward walk test (seconds and steps), and the 6-minute walking test (distance in meters). Balance tests including standing heels together, heels with one foot length apart, and feet together; with eyes open or closed; on floor and foam cushion, tandem stance and one-leg stance were evaluated. Cognitive function was assessed by a trained neurologist using Bingley’s visual memory and Identical forms tests [2, 11]. On day 2 at 9:00 AM, an LP was performed with the patients in the lateral recumbent position, followed by measurement of opening pressure and withdrawal of 50 mL of cerebrospinal fluid [11]. The post-tap physiotherapy and cognitive assessment were conducted on day 2 at 2:00 PM, using the same standardized procedure.

The CSF TT was considered positive based on a holistic evaluation during multiprofessional team conferences. A positive result was typically determined when significant improvements were observed in more than one test, supported by video documentation of changes in gait quality and performance.

### Statistical analysis

Descriptive statistics for continuous variables are presented as means and standard deviations (SD) or as medians and ranges, as appropriate. Categorical variables are presented as counts and percentages. Baseline comparison between the NoTT and TT groups were performed using the Mann–Whitney U test for continuous variables and Fisher’s exact test for categorical variables.

Baseline MRI characteristics were compared between NoTT and TT groups using the independent sample t-test for continuous variables, Mann-Whitney U-test for ordinal variables, or the Pearson’s Chi-square test for categorical variables.

The overall proportions of total iNPH Scale (improved, unchanged and deteriorated) between NoTT and TT groups were compared using Pearson’s Chi-square test. Differences in proportions between groups were evaluated using Wald tests and are presented as absolute percentage differences with 95% confidence intervals (CIs).

Within-group pre-operative to postoperative changes were analyzed using the paired *t*-test. Between-group differences in pre-operative values, postoperative values, and pre-operative to postoperative changes in iNPH Scale scores were analyzed using Welch’s analysis of covariance (ANCOVA), adjusting for clinically significant baseline differences, specifically time from diagnosis to surgery and the presence of concurrent neurological disease. Analyses of postoperative values and changes were additionally adjusted for the corresponding pre-operative value. Results are presented as mean differences with 95% CIs.

All tests were two-tailed and conducted at the 5% significance level. Statistical analyses were conducted using SAS/STAT^®^ software, version 9.4 (SAS Institute Inc., Cary, NC, USA) and IBM SPSS Statistics, version 28.0.0 (IBM Corp, Armonk, NY, USA).

## Results

### Baseline characteristics and preoperative outcomes

**Fig. 1:**
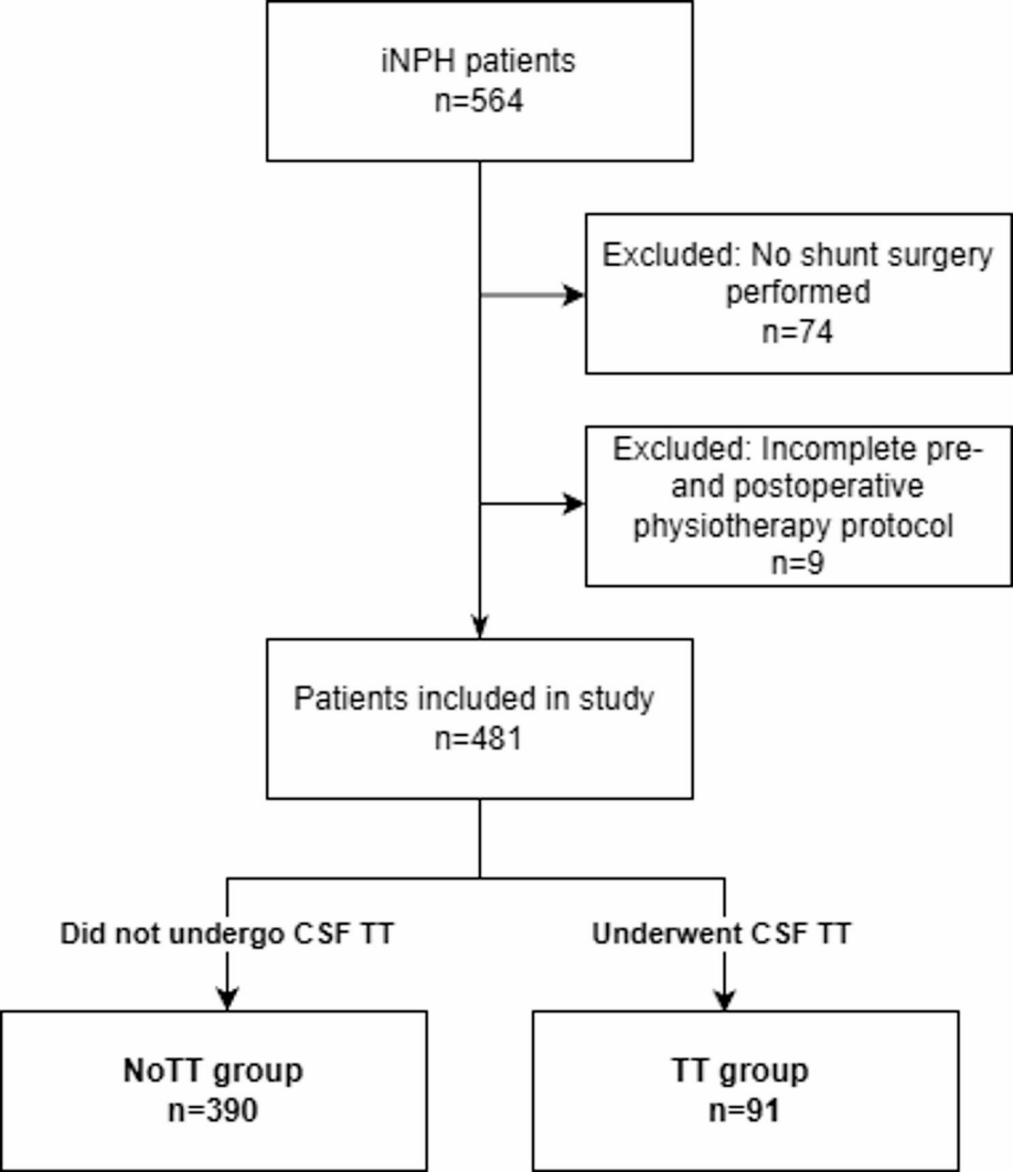
Flowchart of patients included in the study

A total of 564 iNPH patients were eligible for inclusion. Of these, 481 patients met inclusion criteria including 91 patients who underwent CSF TT (TT), and 390 patients who were directly referred to shunt surgery (NoTT), Fig. 1.

In the whole sample, mean age was 76 years, 63% were males, and the postoperative follow-up was median 5.0 (IQR 4–8) months. The TT group had a higher prevalence of concurrent neurological disease (36% vs. 11%, *p* < 0.001) and a longer median waiting time from diagnosis to shunt surgery (198 days vs. 126 days, *p* < 0.001), otherwise, no significant differences between the groups were seen, Table 1.

**Table 1:** Group comparisons of baseline data of patients who were shunted without a previous CSF tap test (NoTT) and those who were shunted following a positive CSF tap test (TT)

	NoTT (*n* = 390)		TT (*n* = 91)		*p*-value
Age, years, median (range)	*n* = 390	76 (72–80)	*n* = 91	76 (72–80)	0.50
Sex M: F (M%: F%)	*n* = 390	250:140 (64%:36%)	*n* = 91	52:39 (57%:43%)	0.21
BMI, kg/m^2^, median (range)	*n* = 370	27 (24–29)	*n* = 88	26 (24–28)	0.06
**Comorbidities**,** n (%)**					
Hypertension	*n* = 388	240 (62%)	*n* = 91	61 (67%)	0.34
Diabetes	*n* = 387	102 (26%)	*n* = 91	24 (26%)	0.98
Cardiovascular	*n* = 387	115 (30%)	*n* = 91	29 (32%)	0.67
Hyperlipidemia	*n* = 256	110 (43%)	*n* = 72	33 (46%)	0.64
Ischemic heart disease	*n* = 260	41 (16%)	*n* = 73	9 (12%)	0.47
Other heart disease	*n* = 278	53 (19%)	*n* = 76	18 (24%)	0.36
Cerebrovascular disease	*n* = 277	51 (18%)	*n* = 77	22 (29%)	0.056
Other concurrent neurological disease	*n* = 221	24 (11%)	*n* = 60	22 (37%)	**< 0.001**
Disease duration, months, median (range)	*n* = 372	36 (24–60)	*n* = 88	39.5 (24–60)	0.20
Time from diagnosis to surgery in days, median (range)	*n* = 389	126 (81–185)	*n* = 91	198 (142–263)	**< 0.001**
**Shunt type**,** n (%)**					
Ventriculoperitoneal	*n* = 389	378 (97%)	*n* = 91	85 (93%)	0.19
Ventriculoatrial		11 (3%)		6 (7%)	

Abbreviations: M, Male; F, Female; BMI, Body Mass Index

Baseline characteristics were compared using Fisher’s exact test for categorical variables and the Mann-Whitney U test for continuous variables

Among the 46 patients with concurrent neurological disease in the whole sample, the distribution were; Alzheimer’s Disease (26.1%), vascular cognitive disorder (17.4%), Parkinson’s Disease (4.4%), Corticobasal degeneration (2.2%), and other concurrent neurological diseases (50%). The remaining concurrent neurological diseases (50%) (*n* = 1 if not otherwise specified) registered in NoTT patients were Guillain-Barré syndrome, Huntington’s disease, spinal stenosis, peripheral neuropathy (*n* = 2), earlier alcohol abuse (*n* = 2), congenital cerebral palsy, neurodegenerative memory disorder, scoliosis, unspecified (*n* = 5). In the TT group: earlier alcohol abuse, multiple sclerosis (*n* = 2), parkinsonism, lumbar spinal stenosis, other neurodegenerative disorder and unspecified conditions (*n* = 3). The detailed breakdown of comorbidities is presented in Supplementary Table 1.

Baseline MRI features were available for a subset of the cohort. No significant differences between NoTT and TT patients were found in any of the MRI measures (Table 2).

**Table 2:** Baseline MRI measures comparison of NoTT and TT groups

		NoTT		TT		*p*-value
Evans’ index, mean (SD)		*n* = 164	0.370 (0.04)	*n* = 53	0.372 (0.04)	0.74^a^
Callosal angle (°), mean (SD)		*n* = 161	70.90 (16.2)	*n* = 54	74.31 (16.9)	0.20^a^
Temporal horn width (mm), mean (SD)		*n* = 122	7.25 (2.0)	*n* = 41	7.85 (2.7)	0.13^a^
THC, yes/no (% yes)		*n* = 165	105/60 (66)	*n* = 56	32/24 (57)	0.43^b^
Dilated Sylvian fissures, yes/no (% yes)		*n* = 164	132/32 (80)	*n* = 57	40/17 (70)	0.11^b^
DESH, yes/no (% yes)		*n* = 161	96/65 (60)	*n* = 56	28/28 (50)	0.21^b^
Infarctions, yes/no (% yes)		*n* = 151	34/117 (23)	*n* = 52	11/41 (21)	0.84^b^
Fazekas scale, n (%)	Grade 0	*n* = 150	5 (3.3)	*n* = 53	2 (3.8)	0.64^c^
	Grade 1		35 (23.3)		14 (26.4)	
	Grade 2		63 (42)		17 (32.1)	
	Grade 3		47 (31.3)		20 (37.7)	

Abbreviations: THC, Tight high convexity sulci; DESH, Disproportionally enlarged subarachnoid space hydrocephalus (concurrent presence of both THC and dilated Sylvian fissures)

^a^ Independent sample t-test, ^b^ Chi-square test, ^c^ Mann-Whitney U test

### Postoperative outcomes: improved, unchanged, deteriorated

Overall distribution of postoperative outcome categories differed significantly between NoTT and TT (*p* = 0.020). In the study cohort, 68.7% of the patients were deemed overall improved postoperatively in total iNPH Scale score. Overall improvement was observed in 70.9% of NoTT group compared to 58.6% of the TT group (percentage point difference = − 12.3; 95% CI = − 24.3 to − 0.3; *p* = 0.044), whereas postoperative deterioration was significantly twice as common among the TT patients than in the NoTT patients (22.9% vs. 10.7%; percentage point difference = 12.1; 95 CI = 3.5, 20.7, *p* = 0.006). Similar proportions of patients in both groups remained unchanged (NoTT = 18.4%, TT = 18.6%; percentage point difference = 0.2; 95% CI= − 9.9, 10.2; *p* = 0.97) (Table 3).

**Table 3:** Distribution of postoperative outcomes (improved, unchanged, deteriorated) according to change in total iNPH Scale score following shunt surgery in NoTT and TT patients

	All (*n* = 396)	NoTT (*n* = 326)	TT (*n* = 70)	Percentage point difference (TT vs. NoTT) (95% CI)	*p*-value
Improved, *n* (%)	272 (68.7)	231 (70.9)	41 (58.6)	−12.3 (− 24.3, − 0.3)	0.044
Unchanged, n (%)	73 (18.4)	60 (18.4)	13 (18.6)	0.2 (− 9.9, 10.2)	0.97
Deteriorated, n (%)	51 (12.9)	35 (10.7)	16 (22.9)	12.1 (3.5, 20.7)	**0.006**

Improved and deteriorated are defined as a ≥ 5-point increase or decrease, respectively, in the total Gothenburg iNPH Scale score; unchanged was defined as a change between − 4 and + 4 points

Overall differences in postoperative outcome categories between groups were analyzed using Pearson’s Chi-square test (*p* = 0.020)

Percentage point differences with corresponding 95% confidence intervals (CI) and *p*-values were calculated using Wald tests

NoTT: patients who underwent shunt surgery without prior cerebrospinal fluid tap testing

TT: patients who underwent shunt surgery following a positive cerebrospinal fluid tap test

**Fig. 2:**
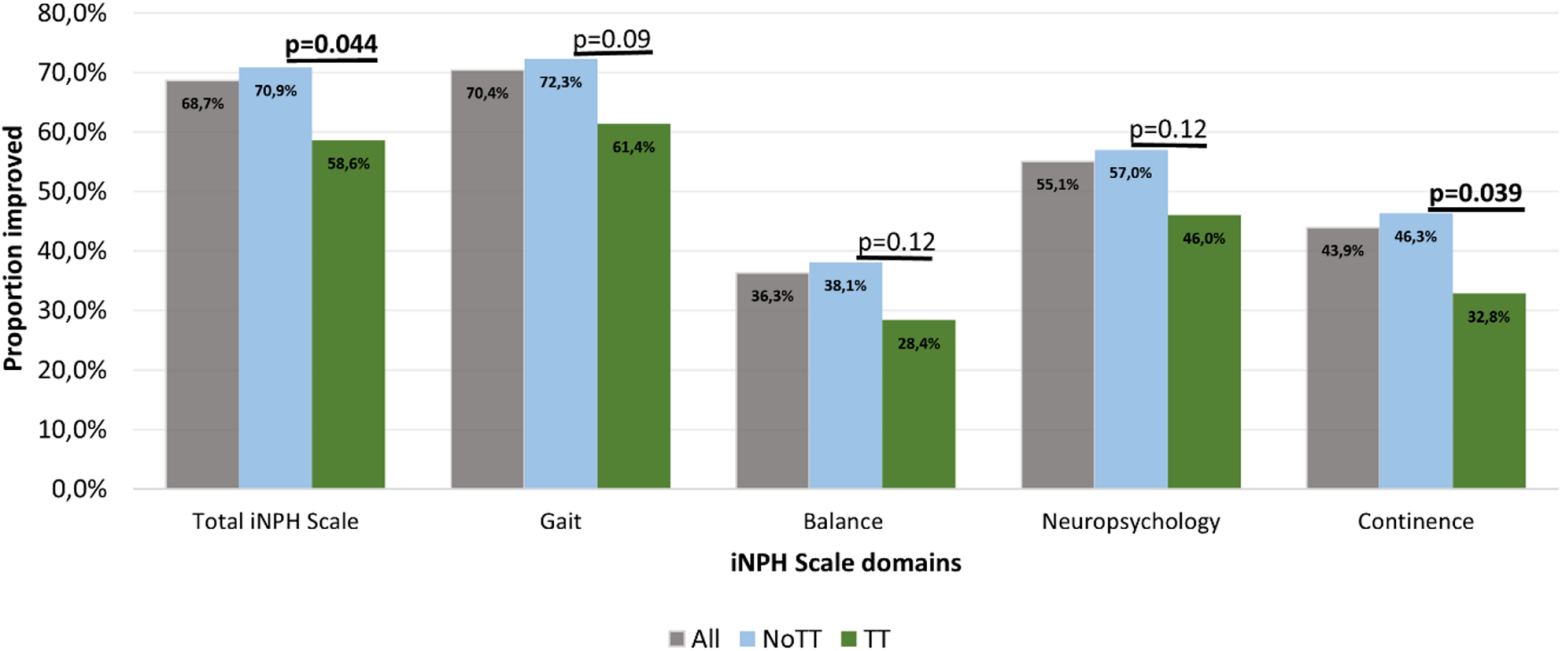
Proportion of patients improved (≥ 5 points increase) in total iNPH Scale score, and in gait, balance, neuropsychology and continence domains (bars) with significance levels of comparison between NoTT and TT groups. Linear regression was used to calculate rate difference between the groups

Improved rates in the iNPH Scale domains are shown in Fig. 2: continence improvement was more common among NoTT patients (46% vs. 33%, *p* = 0.039). The detailed comparison of the proportion of shunt responders (5-point or more increase) across all iNPH Scale domain scores are presented in Supplementary Table 2.

### Postoperative changes in iNPH Scale scores, mRS and MMSE in NoTT and TT patients

Significant increases in total iNPH Scale scores were seen in both groups (NoTT group mean change = 11.8; 95% CI = 10.1, 13.6; TT group mean change = 7.0; 95% CI 3.5, 10.6; *p* < 0.001, for both).

Significant improvements were seen in all iNPH domain Scale scores and mRS in NoTT patients, whereas TT patients were improved in gait and mRS (*p* < 0.001) (Table 4).

After adjustments for corresponding baseline iNPH Scale scores, as well as concurrent neurological conditions and time from diagnosis to surgery, mean change in the continence score was significantly greater in the NoTT group (*p* = 0.035) (Table 4). Detailed results of unadjusted between-group comparisons, and adjusted postoperative assessments of iNPH scores, mRS, MMSE are presented in Supplementary Tables 3 and Supplementary 4, respectively.

**Table 4:** iNPH Scale total and domain scores, modified Rankin Scale and MMSE before and after shunt surgery in NoTT and TT patients

	NoTT (*n* = 390)			TT (*n* = 91)			Difference between groups			
	Preop	Postop	*p*-value	Preop	Postop	*p*-value	Preop adjusted mean difference (95% CI)	*p*-value	Adjusted difference of mean change (95% CI)	*p*-value
***iNPH Scale***										
Total	53.2 (15.8)	65.2 (19.5)	**< 0.001**	50.7 (15.9)	57.9 (19.4)	**< 0.001**	−0.5 (− 5.5, 4.5)	0.83	−2.9 (− 8.6, 2.8)	0.32
Gait	45.7 (23.5)	64.3 (26.5)	**< 0.001**	45.2 (22.4)	57.8 (27)	**< 0.001**	2.8 (− 4.3, 10)	0.43	−4.3 (− 11.8, 3.1)	0.25
Balance	64.2 (17.7)	69.9 (17.8)	**< 0.001**	61.3 (19.2)	62.8 (22.4)	0.94	−2 (− 8, 4)	0.52	−0.8 (− 9, 7.5)	0.85
Neuropsychology	54.7 (18.9)	60.9 (20.4)	**< 0.001**	46.8 (21.1)	50.7 (25.1)	0.21	−4 (− 10.3, 2.4)	0.22	0.2 (− 5.2, 5.7)	0.94
Continence	55.7 (26.4)	68.9 (27.7)	**< 0.001**	55.2 (26.2)	58.8 (28.4)	0.14	−2.8 (− 11.1, 5.5)	0.51	−8.7 (− 16.7, − 0.6)	**0.035**
**mRS**	2.6 (0.8)	2.1 (1)	**< 0.001**	2.9 (0.9)	2.4 (1)	**< 0.001**	0.3 (− 0.01, 0.5)	0.064	−0.1 (− 0.4, 0.2)	0.55
**MMSE**	25.6 (3.6)	25.9 (3.4)	0.52	24.2 (4.4)	25 (4)	0.35	−0.8 (− 2, 0.5)	0.22	0.3 (− 0.8, 1.4)	0.62

Pre- and postoperative values presented as mean (standard deviation). Within-group comparisons of pre-operative to postoperative values were conducted using the paired *t*-test

Differences between groups are presented as adjusted mean differences with 95% confidence intervals (CIs), adjusted for time from diagnosis to surgery and the presence of concurrent neurological disease. Analyses of change values were additionally adjusted for the corresponding pre-operative value. All between-group differences were evaluated using Welch’s analysis of covariance (ANCOVA)

Abbreviations: mRS, modified Rankin Score; MMSE, Mini-Mental State Examination

## Discussion

We report baseline characteristics and postoperative clinical outcomes in a large, real-life cohort of consecutive patients diagnosed with iNPH who underwent shunt surgery based solely on typical clinical symptoms and characteristic MRI findings (NoTT) or after a supplementary positive tap test (TT). Both groups demonstrated significant postoperative improvement. However, after adjustment for confounders, NoTT patients showed more favorable outcomes in continence. While other baseline characteristics were comparable, concurrent neurological disorders were more frequent and the interval from diagnosis to surgery was significantly longer in the TT group which probably contributes to this difference. This study provides evidence that patients diagnosed with iNPH can be selected for shunt surgery based on assessment of clinical symptoms and MRI, without a prior tap test.

In this consecutive cohort of 481 patients evaluated through a multiprofessional, team-based diagnostic approach, 81% underwent shunt surgery based solely on characteristic clinical symptoms and MRI features, without an additional predictive test. This high reliance on symptomatology and MRI aligns with the European multicenter study [5] and the current Japanese iNPH Guidelines [4], and indicates that even a majority of patients can be successfully selected for surgery based on this algorithm. We consider a comprehensive evaluation of iNPH symptomatology—incorporating physiotherapy and neuropsychological assessments, along with a critical review of both the iNPH state and its differential diagnoses—essential for establishing a reliable diagnosis and prognosis, particularly when comorbid neurological disorders are present. A key finding in our study is the radiological comparability between the two groups. Despite different diagnostic pathways, the NoTT and TT groups showed no significant differences in Evans’ index, callosal angle or DESH features, or regarding signs of comorbid cerebrovascular disease. Our multidisciplinary team achieved an improvement rate comparable to that reported by Hashimoto et al. [26], whose findings demonstrated that the presence of DESH features carries a high positive predictive value for shunt response.

Given the low sensitivity of the CSF TT [10–14], which carries a risk of excluding patients who could benefit from treatment, our selection process (in which only 19% required a CSF TT) likely results in a greater proportion of patients with iNPH receiving appropriate intervention than would be achieved if shunt decisions were based on CSF TT results alone [12]. This reasoning may also apply to other predictive tests, such as extended lumbar drainage or lumbar infusion testing [8, 30].

Baseline distributions regarding age, sex, BMI, prevalent vascular risk factors, and global cognitive functions were similar across NoTT and TT groups whereas a higher proportion of concurrent neurological diseases was found in TT patients. Previous studies in iNPH have shown that comorbidities influence outcomes and that a better postoperative improvement is associated with a lower comorbidity index, including reduced burden of vascular encephalopathy, PD, or AD [31, 32]. This aligns with our clinical experience, and our present results implies that CSF TT is more often deemed necessary in cases with signs of neurological comorbidity. Our data on concurrent neurological diseases were diverse. However, limited both in detail and number of observations and did not allow a more detailed analysis of this association.

Our overall positive responder rate of 68.7% aligns with previously reported outcomes [5, 24, 33] and is higher than in a large national cohort from the Swedish Hydrocephalus Quality Registry [34], reinforcing the clinical applicability of our routine. Nine out of ten patients in NoTT group (89.3%) were either postoperatively improved or unchanged, and significantly better outcomes were seen compared to the TT group: postoperative improvement in total and all four iNPH Scale domains and mRS were observed in NoTT, whereas only gait and mRS improved in TT patients. Interestingly, postoperative deterioration was more common among TT patients, possibly due to the more abundant concurrent neurological disorders. These differences together with the lower response rate of 58.6% in the TT group indicate that this selected group of patients have a poorer prognosis in spite of a positive CSF TT and that careful assessment of factors predictive of outcome such as concurrent neurological disease may be crucial. When adjusted for concurrent neurological disorder and surgical delay, improvement in continence domain remained significantly better in NoTT patients suggesting that both factors contributed to the worse outcomes seen in TT patients.

Our diagnostic procedure, using the CSF TT as an additional test, led to a significantly longer surgical delay with a median waiting time until surgery of 72 days longer in the TT group. This prolonged delay may have affected outcomes as longer waiting times have been shown to worsen prognosis [35, 36]. We believe that when a predictive test is deemed necessary, it should be performed without delay to avoid poorer outcomes, a practice that has now been implemented in our service.

Our CSF TT protocol, which is a modified version of the original report by Wikkelsö et al. [11], includes a multi-domain physiotherapy-based test battery designed to maximize sensitivity. By evaluating a combination of gait, static and dynamic balance, we believe this approach provides a more comprehensive functional profile than reliance on a single measure (e.g. the 10-meter walking test alone). In our practice, CSF TT is considered positive in a holistic approach during multiprofessional team conferences when significant improvements is observed in more than one test, supported by video documentation of changes in gait performance. In a recent publication comparing these physiotherapy-based tests, we conclude that including gait assessments in direction and capacity, such as 3MBW and 6MWT, together with tests of postural control increases the sensitivity to change after CSF removal in iNPH patients [37]. While our methodology prioritizes clinical sensitivity, the absence of a universally defined cut-off for what constitutes a clinically meaningful change represents a limitation and may affect comparability across centers. Nevertheless, our findings reinforce the importance of a rigorous, multiprofessional team-based quantitative assessment to navigate the diagnostic uncertainty of iNPH and optimizing surgical outcomes.

In our center, a lumbar infusion test is used for selected cases to strengthen the diagnosis and indication for surgery. For the purposes of this study, only patients who were operated on without the use of a predictive test or following a positive CSF TT were included in the NoTT and TT groups to ensure homogeneity.

We acknowledge the risk of denying treatment to complex patients who may fail the CSF TT, despite having a strong clinical and/or MRI suspicion of iNPH. In this real-world setting, comprising patients diagnosed 2017–2023, clear support of a positive test result was required for shunting in the CSF TT patients. Adding a second CSF TT attempt, infusion test or extended lumbar drainage to the diagnostic algorithm in all patients who do not demonstrate a significant response to CSF TT but still present with clinical indicators of iNPH, as recently suggested by Panciani et al. [38] for the Italian Neurosurgical Society, is an interesting approach to minimize risk of false negatives that should be evaluated in future studies.

### Strengths and limitations

Strengths of this study include the large, consecutive, real-life representative iNPH patient cohort, the detailed assessment of clinical symptoms and outcomes after 5 months, and the utilization of a multiprofessional team-based setting for diagnosis and treatment in a specialized iNPH center according to established iNPH guidelines.

Some limitations should be acknowledged. While we have included key MRI parameters for a subset of the study cohort, a limitation remains in that these data were not available for the entire study population, although all patients met International Guidelines MRI criteria for iNPH diagnosis at the time of their visits. Our holistic approach with lack of standardized thresholds for significant improvement in the included gait and balance tests, e.g. a 10% improvement in 10-meter walk time, as well as a composite threshold for a positive CSF TT limits the reproducibility of the study. Another limitation is the documented risk that a reliance on a positive CSF TT or lumbar infusion test could exclude several patients who would otherwise benefit from effective treatment [12]. Further, a detailed description of all concurrent neurological diseases and results of baseline CSF biomarker concentrations were not available. Therefore, we could not analyze in detail the prognostic role of different neurological comorbidities or characterize the CSF biomarker profile of the CSF TT group. Finally, the 5-month follow-up in our study may be rather short for a progressive neurodegenerative condition and does not exclude differences in long-term outcomes. This timeframe was chosen to capture clinical benefit from shunt surgery, minimizing age-related declines, or progression of comorbidities. While the 5-month follow-up captured the initial clinical response to shunting, future longitudinal studies are warranted to determine long-term improvement rates in these two cohorts.

## Conclusions

This study shows that even a majority of iNPH patients can be selected for shunt surgery through a multidisciplinary team consensus without a predictive test and still achieve favorable postoperative outcomes comparable to those of patients shunted after a positive CSF TT in the routine clinical setting. The less favorable outcomes seen in the TT group, indicating that patients with diagnostic uncertainty have worse outcomes even with a positive tap test, may at least partly be explained by the higher prevalence of neurological comorbidity, and by the longer delay from diagnosis to surgery. We propose that, following a comprehensive evaluation of clinical symptoms and MRI findings, the CSF tap test should be selectively applied to patients with suspected iNPH and concurrent neurological disorders, considering addition of another predictive test in tap test negative cases, with careful measures in place to prevent clinically significant delays to surgery.

## Supplementary Information

Below is the link to the electronic supplementary material.

Supplementary Material 1

## Data Availability

According to Swedish regulations, the dataset generated within this study’s framework cannot be made publicly available for ethical and legal reasons. The research data can be made available on reasonable request to the corresponding author.

## References

[1] Relkin N, Marmarou A, Klinge P, Bergsneider M, McL Black P. Diagnosing idiopathic normal-pressure hydrocephalus. Neurosurgery. Volume 57. Oxford University Press; 2005. pp. S24–216. 10.1227/01.NEU.0000168185.29659.C5. 10.1227/01.neu.0000168185.29659.c5 16160425

[2] Agerskov S, Hellström P, Andrén K, Kollén L, Wikkelsö C, Tullberg M. The phenotype of idiopathic normal pressure hydrocephalus-a single center study of 429 patients. J Neurol Sci Elsevier B V. 2018;391:54–60. 10.1016/j.jns.2018.05.022. 10.1016/j.jns.2018.05.022 30103972

[3] Constantinescu C, Wikkelsø C, Westman E, Ziegelitz D, Jaraj D, Rydén L, et al. Prevalence of Possible Idiopathic Normal Pressure Hydrocephalus in Sweden: A Population-Based MRI Study in 791 70-Year-Old Participants. Neurology. Lippincott Williams and Wilkins; 2024. p. 102. 10.1212/WNL.0000000000208037. 10.1212/WNL.0000000000208037 PMC10962905 38165321

[4] Nakajima M, Yamada S, Miyajima M, Ishii K, Kuriyama N, Kazui H, et al. Guidelines for management of idiopathic normal pressure hydrocephalus (Third edition): Endorsed by the Japanese society of normal pressure hydrocephalus. Neurol Med Chir (Tokyo) Japan Neurosurgical Soc. 2021;61:63–97. 10.2176/nmc.st.2020-0292. 10.2176/nmc.st.2020-0292 PMC7905302 33455998

[5] Klinge P, Hellström P, Tans J, Wikkelsø C. One-year outcome in the European multicentre study on iNPH. Acta Neurol Scand. 2012;126:145–53. 10.1111/j.1600-0404.2012.01676.x. 22571428 10.1111/j.1600-0404.2012.01676.x

[6] Tullberg M, Persson J, Petersen J, Hellström P, Wikkelsø C, Lundgren-Nilsson Å. Shunt surgery in idiopathic normal pressure hydrocephalus is cost-effective—a cost utility analysis. Acta Neurochir (Wien). Springer-Verlag Wien; 2018;160:509–18. 10.1007/s00701-017-3394-7. 10.1007/s00701-017-3394-7 PMC5807454 29150794

[7] Pearce RKB, Gontsarova A, Richardson D, Methley AM, Watt HC, Tsang K et al. Shunting for idiopathic normal pressure hydrocephalus. Cochrane Database of Systematic Reviews. John Wiley and Sons Ltd; 2024;2024. 10.1002/14651858.CD014923.pub2. 10.1002/14651858.CD014923.pub2 PMC11301990 39105473

[8] Kahlon B, Sundbärg G, Rehncrona S. Comparison between the lumbar infusion and CSF tap tests to predict outcome after shunt surgery in suspected normal pressure hydrocephalus [Internet]. J Neurol Neurosurg Psychiatry. 2002. www.jnnp.com. 10.1136/jnnp.73.6.721 PMC1757331 12438477

[9] Halperin JJ, Kurlan R, Schwalb JM, Cusimano MD, Gronseth G, Gloss D. Practice guideline: Idiopathic normal pressure hydrocephalus: Response to shunting and predictors of response Report of the Guideline Development, Dissemination, and Implementation Subcommittee of the American Academy of Neurology [Internet]. 2015. https://www.neurology.org. 10.1212/WNL.0000000000002193 PMC4676757 26644048

[10] Marmarou A, Bergsneider M, Klinge P, Relkin N, Black PML. INPH guidelines, part III: The value of supplemental prognostic tests for the preoperative assessment of idiopathic normal-pressure hydrocephalus. Neurosurgery. 2005. 10.1227/01.NEU.0000168184.01002.60. 16160426 10.1227/01.neu.0000168184.01002.60

[11] Wikkelsö C, Andersson H, Blomstrand C, Lindqvist G, Svendsen P. Predictive value of the cerebrospinal fluid tap-test. Acta Neurol Scand. 1986;73:566–73. 10.1111/j.1600-0404.1986.tb04601.x. 3751498 10.1111/j.1600-0404.1986.tb04601.x

[12] Wikkelsø C, Hellström P, Klinge PM, Tans JTJ. The European iNPH Multicentre Study on the predictive values of resistance to CSF outflow and the CSF Tap Test in patients with idiopathic normal pressure hydrocephalus. J Neurol Neurosurg Psychiatry BMJ Publishing Group. 2013;84:562–8. 10.1136/jnnp-2012-303314. 10.1136/jnnp-2012-303314 23250963

[13] Ishikawa M, Hashimoto M, Mori E, Kuwana N, Kazui H. The value of the cerebrospinal fluid tap test for predicting shunt effectiveness in idiopathic normal pressure hydrocephalus. Fluids Barriers CNS. 2012;9. 10.1186/2045-8118-9-1. 10.1186/2045-8118-9-1 PMC3293050 22239832

[14] Hamilton MG, Williams MA, Edwards S, Tullberg M. Guidelines for diagnosis and management of idiopathic normal pressure hydrocephalus. Neurosurg Clin N Am. W.B. Saunders; 2025. p. 199–205. 10.1016/j.nec.2024.12.006. 10.1016/j.nec.2024.12.006 40054973

[15] Mori E, Ishikawa M, Kato T, Kazui H, Miyake H, Miyajima M, et al. iNPH guideline guidelines for management of idiopathic normal pressure hydrocephalus: 2nd Edition. Neurol Med Chir (Tokyo); 2012. 10.2176/nmc.52.775 23183074

[16] Andersson J, Rosell M, Kockum K, Söderström L, Laurell K. Challenges in diagnosing normal pressure hydrocephalus: Evaluation of the diagnostic guidelines. eNeurologicalSci Elsevier B V. 2017;7:27–31. 10.1016/j.ensci.2017.04.002. 10.1016/j.ensci.2017.04.002 PMC5746061 29302622

[17] Park HY, Park CR, Suh CH, Kim MJ, Shim WH, Kim SJ. Prognostic utility of disproportionately enlarged subarachnoid space hydrocephalus in idiopathic normal pressure hydrocephalus treated with ventriculoperitoneal shunt surgery: A systematic review and meta-analysis. American Journal of Neuroradiology. American Society of Neuroradiology; 2021. pp. 1429–36. 10.3174/ajnr.A7168. 10.3174/ajnr.A7168 PMC8367621 34045302

[18] Luciano MG, Williams MA, Hamilton MG, Katzen HL, Dasher NA, Moghekar A, et al. A Randomized Trial of Shunting for Idiopathic Normal-Pressure Hydrocephalus. New Engl J Med Mass Med Soc. 2025. 10.1056/nejmoa2503109. 10.1056/NEJMoa2503109 PMC12682072 40960253

[19] Williams MA, Relkin NR, Summary P. Neurology ^®^ Clinical Practice Diagnosis and management of idiopathic normal-pressure hydrocephalus [Internet]. 2013. www.neurology.org/cp. 10.1212/CPJ.0b013e3182a78f6b PMC3806933 24175154

[20] Constantinescu C, Ziegelitz D, Wikkelsø C, Kern S, Jaraj D, Rydén L, et al. MRI markers of idiopathic normal pressure hydrocephalus in a population study with 791 participants: Exploring reference values and associations. Neuroradiology Journal. SAGE Publications Inc.; 2024. 10.1177/19714009241303132. 10.1177/19714009241303132 PMC11626555 39648970

[21] Andrén K, Wikkelsø C, Tisell M, Hellström P. Natural course of idiopathic normal pressure hydrocephalus. J Neurol Neurosurg Psychiatry BMJ Publishing Group. 2014;85:806–10. 10.1136/jnnp-2013-306117. 10.1136/jnnp-2013-306117 24292998

[22] Hellström P, Klinge P, Tans J, Wikkelsø C. A new scale for assessment of severity and outcome in iNPH. Acta Neurol Scand. 2012;126:229–37. 10.1111/j.1600-0404.2012.01677.x. 22587624 10.1111/j.1600-0404.2012.01677.x

[23] Rydja J, Kollén L, Hellström P, Owen K, Lundgren Nilsson Å, Wikkelsø C, et al. Physical exercise and goal attainment after shunt surgery in idiopathic normal pressure hydrocephalus: a randomised clinical trial. Fluids Barriers CNS BioMed Cent Ltd. 2021;18. 10.1186/s12987-021-00287-8. 10.1186/s12987-021-00287-8 PMC8607575 34809666

[24] Andrén K, Wikkelsø C, Laurell K, Kollén L, Hellström P, Tullberg M. Symptoms and signs did not predict outcome after surgery: a prospective study of 143 patients with idiopathic normal pressure hydrocephalus. J Neurol Springer Sci Bus Media Deutschland GmbH. 2024;271:3215–26. 10.1007/s00415-024-12248-w. 10.1007/s00415-024-12248-w PMC11136756 38438818

[25] Wikkelsø C, Andersson H, Lindberg S, Blomstrand C. “Shuntography” - A radionuclide scanning method for evaluation of cerebrospinal fluid shunt patency. Nucl Med Commun. 1983;4.

[26] Hashimoto M, Ishikawa M, Mori E, Kuwana N. Diagnosis of idiopathic normal pressure hydrocephalus is supported by MRI-based scheme: a prospective cohort study. Cerebrospinal Fluid Res. 2010;7. 10.1186/1743-8454-7-18. 10.1186/1743-8454-7-18 PMC2987762 21040519

[27] Kockum K, Virhammar J, Riklund K, Söderström L, Larsson EM, Laurell K. Standardized image evaluation in patients with idiopathic normal pressure hydrocephalus: consistency and reproducibility. Neuroradiol Springer Verlag. 2019;61:1397–406. 10.1007/s00234-019-02273-2. 10.1007/s00234-019-02273-2 PMC6848037 31399851

[28] Broderick JP, Adeoye O, Elm J. Evolution of the modified rankin scale and its use in future stroke trials. Stroke. Lippincott Williams and Wilkins; 2017. pp. 2007–12. 10.1161/STROKEAHA.117.017866. 10.1161/STROKEAHA.117.017866 PMC5552200 28626052

[29] Crum RM, Anthony JC, Bassett SS, Folstein MF. Population-based norms for the mini-mental state examination by age and educational level [Internet]. http://jama.jamanetwork.com/. 8479064

[30] El Ahmadieh TY, Wu EM, Kafka B, Caruso JP, Neeley OJ, Plitt A, et al. Lumbar drain trial outcomes of normal pressure hydrocephalus: A single-center experience of 254 patients. J Neurosurg Am Association Neurol Surg. 2020;132:306–12. 10.3171/2018.8.JNS181059. 10.3171/2018.8.JNS181059 30611143

[31] Malm J, Graff-Radford NR, Ishikawa M, Kristensen B, Leinonen V, Mori E, et al. Influence of comorbidities in idiopathic normal pressure hydrocephalus - research and clinical care. A report of the ISHCSF task force on comorbidities in INPH. Fluids Barriers CNS. 2013;10. 10.1186/2045-8118-10-22. 10.1186/2045-8118-10-22 PMC3689166 23758953

[32] Lemcke J, Meier U. Idiopathic Normal Pressure Hydrocephalus (iNPH) and co-morbidity: An outcome analysis of 134 patients. Acta Neurochir Suppl (Wien). Springer-Verlag Wien; 2012. pp. 255–9. 10.1007/978-3-7091-0956-4_50. 10.1007/978-3-7091-0956-4_50 22327704

[33] Toma AK, Papadopoulos MC, Stapleton S, Kitchen ND, Watkins LD. Systematic review of the outcome of shunt surgery in idiopathic normal-pressure hydrocephalus. Acta Neurochir (Wien). 2013:1977–80. 10.1007/s00701-013-1835-5. 10.1007/s00701-013-1835-5 23975646

[34] Sundström N, Rydja J, Virhammar J, Kollén L, Lundin F, Tullberg M. The timed up and go test in idiopathic normal pressure hydrocephalus: a Nationwide Study of 1300 patients. Fluids Barriers CNS. BioMed Cent Ltd. 2022;19. 10.1186/s12987-021-00298-5. 10.1186/s12987-021-00298-5 PMC8750754 35012586

[35] Andrén K, Wikkelsø C, Hellström P, Tullberg M, Jaraj D. Early shunt surgery improves survival in idiopathic normal pressure hydrocephalus. Eur J Neurol Blackwell Publishing Ltd. 2021;28:1153–9. 10.1111/ene.14671. 10.1111/ene.14671 PMC7986742 33316127

[36] Chidiac C, Sundström N, Tullberg M, Arvidsson L, Olivecrona M. Waiting time for surgery influences the outcome in idiopathic normal pressure hydrocephalus — a population-based study. Acta Neurochir (Wien) Springer. 2022;164:469–78. 10.1007/s00701-021-05085-7. 10.1007/s00701-021-05085-7 PMC8854261 34970701

[37] Akar K, Kollén L, Tullberg M, Persson HC. Sensitivity of physiotherapy-based clinical tests in detecting change in gait and balance performance following a 50 mL CSF tap test in idiopathic normal pressure hydrocephalus. Fluids Barriers CNS BioMed Cent Ltd. 2026;23. 10.1186/s12987-026-00776-8. 10.1186/s12987-026-00776-8 PMC12931076 41703573

[38] Panciani PP, Palandri G, Petrella G, Tuniz F, de Bonis P, de Maria L, et al. Idiopathic normal pressure hydrocephalus: a systematic review and a streamlined six-step algorithm endorsed by the Italian Society of Neurosurgery (SINCH). J Neurosurg Sci Edizioni Minerva Med. 2025:92–101. 10.23736/S0390-5616.25.06429-X. 10.23736/S0390-5616.25.06429-X 40045807

