# Supplementary material for: Clinical characteristics and outcomes after shunt surgery in idiopathic normal pressure hydrocephalus with or without prior cerebrospinal fluid tap testing: a single-center follow-up study of 481 patients
Source: Fluids Barriers CNS. 2026 Mar 31;23:53. doi: 10.1186/s12987-026-00802-9 (PMC13045051; doi:10.1186/s12987-026-00802-9)
Supplement: Supplementary file 1 — Supplementary Material 1 [file 12987_2026_802_MOESM1_ESM.docx]

**Clinical Characteristics and Outcomes After Shunt Surgery in Idiopathic Normal Pressure Hydrocephalus With or Without Prior Cerebrospinal Fluid Tap Testing: A Single-Center Follow-up Study of 481 Patients**

**Kardelen Akar, Lena Kollen, Hanna C. Persson, Mats Tullberg**

**Supplementary Table 1:** Distribution of concurrent neurological disorders in both groups

|  | All  (n=46) | NoTT  (n=24) | TT  (n=22) |
| --- | --- | --- | --- |
| Alzheimer's Disease, n (%) | 12 (26.1) | 7 (29.2) | 5 (22.7) |
| Vascular cognitive disorder, n (%) | 8 (17.4) | 1 (4.2) | 7 (31.8) |
| Parkinson's Disease, n (%) | 2 (4.3) | 1 (4.2) | 1 (4.5) |
| Corticobasal degeneration, n (%) | 1 (2.2) | 0 | 1 (4.5) |
| Other, n (%) | 23 (50) | 15 (62.5) | 8 (36.4) |
| *Guillain-Barré syndrome* | *1* | *1* | *0* |
| *Huntington’s Disease* | *1* | *1* | *0* |
| *Spinal stenosis* | *2* | *1* | *1* |
| *Peripheral neuropathy* | *2* | *2* | *0* |
| *Alcohol abuse* | *3* | *2* | *1* |
| *Congenital cerebral palsy* | *1* | *1* | *0* |
| *Neurodegenerative memory disorder* | *1* | *1* | *0* |
| *Scoliosis* | *1* | *1* | *0* |
| *Multiple sclerosis* | *2* | *0* | *2* |
| *Parkinsonism* | *1* | *0* | *1* |
| *Other unspecified condition* | *8* | *5* | *3* |

**Supplementary Table 2** Proportion of postoperatively improved patients defined as an increase of ≥5 iNPH scale score points (total, gait, balance, neuropsychology and continence) in NoTT and TT patients.

|  | **All (n=481)** | **NoTT (n=390)** | **TT (n=91)** | **Rate difference (95% CI)** | **p-value** |
| --- | --- | --- | --- | --- | --- |
| **Total increase of 5 units or more** | | | | | |
| No | 124.0 (31.3%) | 95.0 (29.1%) | 29.0 (41.4%) |  |  |
| Yes | 272.0 (68.7%) | 231.0 (70.9%) | 41.0 (58.6%) | -12.3 (-24.3, -0.3) | **0.044** |
| Missing | 85 | 64 | 21 |  |  |
| **Gait increase of 5 units or more** | | | | | |
| No | 117.0 (29.6%) | 90.0 (27.7%) | 27.0 (38.6%) |  |  |
| Yes | 278.0 (70.4%) | 235.0 (72.3%) | 43.0 (61.4%) | -10.9 (-23.5, 1.7) | 0.090 |
| Missing | 86 | 65 | 21 |  |  |
| **Neuropsychology increase of 5 units or more** | | | | | |
| No | 164.0 (44.9%) | 130.0 (43.0%) | 34.0 (54.0%) |  |  |
| Yes | 201.0 (55.1%) | 172.0 (57.0%) | 29.0 (46.0%) | -10.9 (-24.7, 2.8) | 0.12 |
| Missing | 116 | 88 | 28 |  |  |
| **Balance increase of 5 units or more** | | | | | |
| No | 240.0 (63.7%) | 192.0 (61.9%) | 48.0 (71.6%) |  |  |
| Yes | 137.0 (36.3%) | 118.0 (38.1%) | 19.0 (28.4%) | -9.7 (-22.0, 2.6) | 0.12 |
| Missing | 104 | 80 | 24 |  |  |
| **Continence increase of 5 units or more** | | | | | |
| No | 206.0 (56.1%) | 161.0 (53.7%) | 45.0 (67.2%) |  |  |
| Yes | 161.0 (43.9%) | 139.0 (46.3%) | 22.0 (32.8%) | -13.5 (-26.3, -0.7) | **0.039** |
| Missing | 114 | 90 | 24 |  |  |
| Categorical variables are presented in numbers and percentages. Linear regression was used to calculate rate difference between the groups, which is an estimator for the difference in proportions between groups. **Abbreviations:**CI, confidence interval. | | | | | |

**Supplementary Table 3**: Unadjusted group comparisons of clinical measurements pre- and post-surgery and postoperative change (NoTT vs TT patients)

|  | **Unadjusted Mean difference (95% CI)**  **Between NoTT and TT** | **p-value** |
| --- | --- | --- |
| **Preoperative** | | |
| iNPH Total | -2.45 (-6.13, 1.22) | 0.19 |
| iNPH Gait | -0.52 (-5.74, 4.69) | 0.84 |
| iNPH Balance | -2.89 (-7.26, 1.47) | 0.19 |
| iNPH Neuropsychology | -7.96 (-12.75, -3.17) | **0.001** |
| iNPH Continence | -0.52 (-6.58, 5.53) | 0.86 |
| modified Rankin Scale, score, mean (SD) | 0.29 (0.08, 0.50) | **0.008** |
| MMSE, score, mean (SD) | -1.46 (-2.44, -0.47) | **0.004** |
| **Postoperative** | | |
| iNPH total | -7.25 (-12.32, -2.17) | **0.006** |
| iNPH Gait | -6.56 (-13.60, 0.48) | 0.068 |
| iNPH Balance | -7.11 (-12.93, -1.29) | **0.017** |
| iNPH Neuropsychology | -10.21 (-16.95, -3.48) | **0.003** |
| iNPH continence | -10.10 (-17.70, -2.51) | **0.010** |
| modified Rankin Scale, score, mean (SD) | 0.26 (-0.02, 0.54) | 0.070 |
| MMSE, score, mean (SD) | -0.89 (-1.97, 0.19) | 0.11 |
| **Post-pre change** | | |
| iNPH total | -4.79 (-8.72, -0.87) | **0.017** |
| iNPH Gait | -5.60 (-11.12, -0.09) | **0.046** |
| iNPH Balance | -5.36 (-11.76, 1.05) | 0.10 |
| iNPH Neuropsychology | -3.20 (-7.14, 0.74) | 0.11 |
| iNPH continence | -9.05 (-14.97, -3.14) | **0.003** |
| modified Rankin Scale, score, mean (SD) | 0.03 (-0.25, 0.30) | 0.85 |
| MMSE, score, mean (SD) | 0.30 (-0.67, 1.26) | 0.54 |

**Supplementary Table 4**: Comparison of adjusted postoperative clinical measurements

|  | NoTT | TT | NoTT vs TT | |
| --- | --- | --- | --- | --- |
|  | Postoperative Mean change (95% CI) | Postoperative Mean change (95% CI) | Postoperative Adjusted mean difference (95% CI) | p-value |
| Total | 11.8 (10.1, 13.6) | 7 (3.5, 10.6) | -4 (-10.9, 3) | 0.26 |
| Gait | 18.5 (16.1, 20.9) | 12.9 (7.9, 17.9) | -2.9 (-12.3, 6.6) | 0.55 |
| Balance | 5.6 (3.3, 7.9) | 0.2 (-5.8, 6.2) | -1.4 (-9.7, 7) | 0.74 |
| Neuropsychology | 5.5 (3.9, 7.1) | 2.3 (-1.3, 5.9) | -2.5 (-11.1, 6.1) | 0.57 |
| Continence | 12.9 (10, 15.8) | 3.9 (-1.3, 9.1) | -12.9 (-23.2, -2.6) | **0.015** |
| mRS | -0.5 (-0.6, -0.3) | -0.4 (-0.7, -0.2) | 0.02 (-0.4, 0.4) | 0.91 |
| MMSE | 0.1 (-0.3, 0.5) | 0.4 (-0.5, 1.3) | 0.03 (-1.2, 1.3) | 0.96 |
